# Supplementary material for: Targeted Gene Next-Generation Sequencing Panel in Patients with Advanced Lung Adenocarcinoma: Paving the Way for Clinical Implementation
Source: Cancers (Basel). 2019 Aug 22;11(9):1229. doi: 10.3390/cancers11091229 (PMC6770536; doi:10.3390/cancers11091229)
Supplement: Supplementary file 1 [file cancers-11-01229-s001.pdf]

cancers

Supplementary Materials: Targeted Gene Next-Generation Sequencing Panel in Patients with Advanced Lung Adenocarcinoma: Paving the Way for Clinical Implementation

Table S1. EGFR mutations assessed by Sanger.

EGFR Mutation Annotation Exon	Genomic	Protein	Number of Patients
Exon 19	c.2235_2249del15	p.E746_A750delELREA	8
	c.2236_2250del15	p.E746_A750delELREA	2
	c.2239_2248TTAAGAGAAG > C	p.L747_A750 > P	1
	c.2237_2251del15	p.E746_T751 > A	1
	c.2239_2261del22ins	p.L747_K754delLREATSPK	1
	c.2240_2254del15	p.L747_T751delLREAT	1
	c.2240_2254del15	p.L747_T751delLREAT	1
	c.2240_2257del18	p.L747_P753 > S	2
	c.2248_2276 > CCAAC	p.A750_I759 > PT	1
Exon 20	c.2300_2308dupl p.D770_N771insSVD		1
	c.2310_2311insG	p.D770_N771insG	1
Exon 21	c.2573T > G	p.Leu785Arg	5
Total			22

Table S2. NGS Mutation Annotation and Allelic Frequency of the Experimental cohort.

Mutation	Aminoacid	Clinical Significance	Allelic Frequency
KRAS			
KRAS c.182A > G	p.Gln61Arg	Pathogenic	8.8
KRAS c.35G > A	p.Gly12Asp	Pathogenic	6.1
KRAS c.35G > T	p.Gly12Val	Pathogenic	11.7
KRAS c.35G > T	p.Gly12Val	Pathogenic	27.4
KRAS c.34G > T	p.Gly12Cys	Pathogenic	3.8
KRAS c.35G > T	p.Gly12Val	Pathogenic	6.5
KRAS c.35G > T	p.Gly12Val	Pathogenic	19.9
KRAS c.35G > T	p.Gly12Val	Pathogenic	3.3
KRAS c.34G > T	p.Gly12Cys	Pathogenic	15
KRAS c.38_39delGCinsAA	p.Gly13Glu	Likely pathogenic	14.2
KRAS c.34G > T	p.Gly12Cys	Pathogenic	5.9
KRAS c.35G > T	p.Gly12Val	Pathogenic	20.4
KRAS c.35G > T	p.Gly12Val	Pathogenic	44.7
KRAS c.34G > T	p.Gly12Cys	Pathogenic	5.9
KRAS c.35G > T	p.Gly12Val	Pathogenic	55.2
KRAS c.34G > T	p.Gly12Cys	Pathogenic	17.4
KRAS c.35G > T	p.Gly12Val	Pathogenic	50.6
KRAS c.35G > A	p.Gly12Asp	Pathogenic	22.3
KRAS c.34G > T	p.Gly12Cys	Pathogenic	72.5
KRAS c.35G > T	p.Gly12Val	Pathogenic	26.7
KRAS c.35G > T	p.Gly12Val	Pathogenic	46.3
KRAS c.182A > G	p.Gln61Arg	Pathogenic	0.38
KRAS c.35G > T	p.Gly12Val	Pathogenic	28.6
EGFR			
EGFR c.2240_2257del18	p.L747_P753 > S	Pathogenic	40.0
EGFR c.2238_2252del15	p.L747_T751delLREAT	Pathogenic	27.8
EGFR c.2296_2297insTGGCCAGCG	p.V769_D770insASV	Pathogenic	50.9
EGFR c.2235_2249del15	p.E746_A750delELREA	Pathogenic	74.1
EGFR c.2235_2249del15	p.E746_A750delELREA	Pathogenic	56.5
EGFR c.2248_2276 > CCAAC	p.A750_I759 > PT	Pathogenic	84.5
EGFR c.2573T > G	p.Leu785Arg	Pathogenic	29.2
EGFR c.2235_2249del15	p.E746_A750delELREA	Pathogenic	7.6
EGFR c.2236_2250del15	p.E746_A750delELREA	Pathogenic	11.1
EGFR c.2573T > G	p.Leu785Arg	Pathogenic	18.6

EGFR c.2236_2250del15	p.E746_A750delELREA	Pathogenic	36.7
EGFR c.2573T > G	p.Leu785Arg	Pathogenic	31.3
EGFR c.2240_2254del15	p.L747_T751delLREAT	Pathogenic	96.5
EGFR c.2239_2248TTAAGAGAAG > C	p.L747_A750 > P	Pathogenic	66.0
EGFR c.2240_2257del18	p.L747_P753 > S	Pathogenic	67.2
EGFR c.2235_2249del15	p.E746_A750delELREA	Pathogenic	20.4
EFGR c.2235_2249del15	p.E746_A750delELREA	Pathogenic	13.2
EGFR c.2239_2248TTAAGAGAAG > C	p.L747_A750 > P	Pathogenic	14.0
EGFR c.2573T > G	p.Leu785Arg	Pathogenic	6.3
EGFR c.2573T > G	p.Leu785Arg	Pathogenic	15.3
EGFR c.2369C > T	p.Thr790Met	Pathogenic	0.6
EGFR c.2235_2249del15	p.E746_A750delELREA	Pathogenic	34.6
EGFR c.2235_2249del15	p.E746_A750delELREA	Pathogenic	35.8
EGFR c.2235_2249del15	p.E746_A750delELREA	Pathogenic	31.7
EGFR c.2236_2250del15	p.E746_A750delELREA	Pathogenic	0.17
BRAF			
BRAF c.1799T > A	p.Val600Glu	Pathogenic	36.3
BRAF c.1799T > A	p.Val600Glu	Pathogenic	50.4
BRAF c.1799T > A	p.Val600Glu	Pathogenic	29.1
BRAF c.1799T > A	p.Val600Glu	Pathogenic	10.9
BRAF c.1799T > A	p.Val600Glu	Pathogenic	11.2
TP53			
TP53 c.524G > A	p.Arg175His	Pathogenic	2.13
TP53 c.538G > T	p.Glu180Lys	Likely pathogenic	37.8
TP53 c.839G > A	p.Arg280Lys	Likely pathogenic	13.0
TP53 c.476C > T	p.Ala159Val	Uncertain	39.2
TP53 c.461G > T	p.Gly154Val	Uncertain	24.7
TP53 c.1024C > T	p.Arg342Ter	Pathogenic	21.0
TP53 c.493C > T	p.Gln165Ter	Pathogenic	14.8
PIK3CA			
PIK3CA c.1633G > A	p.Glu545Gln	Pathogenic	27.8
HERBB2			
HER2 c.2310_2311insGCATAC	p.Ala775_Gly776insTyr	Pathogenic	20.0
HERBB4<br>HERBB4 c.1033G > T	Not Found	Unknown	14.9
ALK			
ALK	c.3512T > A	Pathogenic	0.08
STK11			
STK11	p.Gln37ter	Pathogenic	
STK11	p.Glu199Asp	Pathogenic	12.8

Table S3. NGS Mutation Annotation of the Clinical Implementation Cohort.

Mutation	Aminoacid	Allelic Frequency
KRAS		
c.34G > T	p.Gly12Val	28
c.35G > A	p.Gly12Asp	16
c.179G > A	p.Gly60Asp	11
c.34G > T	p.Gly12Cys	10
c.35G > T	p.Gly12Val	3
c.37G > T	p.Gly12Cys	23
c.38G > A	p.Gly13Asp	86
c.35G > C	p.Gly12Ala	28
c.34G > T	p.Gly12Cys	25
c.34G > T	p.Gly12Cys	9
c.34G > T	p.Gly12Cys	32
c.35G > T	p.Gly12Val	21
c.35G > C	p.Gly12Val	24
c.35G > A	p.Gly12Asp	6
c.182A > G	p.Gln61Arg	26
c.34G > T	p.Gly12Cys	44
c.35G > T	p.Gly12Val	8
c.183A > C	p.Gln61His	29
c.35G > A	p.Gly12Asp	31
c.37G > T	p.Gly13Cys	72
c.34G > T	p.Gly12Cys	21
c.35G > A	p.Gly12Asp	19
c.35G > C	p.Gly12Val	16
c.35G > C	p.Gly12Ala	29
c.34G > T	p.Gly12Cys	25
c.35G > A	p.Gly12Asp	6
c.34G > T	p.Gly12Cys	23
c.35G > C	p.Gly12Asp	36
c.34G > T	p.Gly12Cys	48
c.34G > T	p.Gly12Cys	66
c.34G > T	p.Gly12Cys	18

c.35G > A	p.Gly12Asp	20.3
c.35G > T	p.Gly12Val	158
EGFR		
c.2573T > G	p.Leu858Arg	50
c.2235_2249del	p.E746_A750delELREA	
c.2300_2308dup9	p.Ala767_Val769dup	52.4
c.2237_2255delInsT	p.Glu746_Ser752dellnsVal	22
c.2237_2255delInsT	p.Glu746_Ser752dellnsVal	48
c.2314_2319dup	p.Pro772_His773dup	55
c.2573T > G	p.Leu858Arg	22
c.2235_2249del	p.Glu746_Ala750delInsGlnPro	6
c.2310_2311ins3	p.Asp770_Asn771insTyr	25
c.2235_2249del	p.Glu746_Ala750delInsGlnPro	54
c.2237_2248delInsC	p.Glu746_Ala750delInsGlnPro	25
c.2235_2249del	p.Glu746_Ala750del	33
c.240_2254del	p.leu747_Thr751delInsPro	48
c.2239_2250	p.leu747_Thr751delInsPro	38
c.2235_2249del	p.Glu746_Ala750delInsGlnPro	38
c.2303G > T	p.Ser768Ile	12
c.2573T > G	p.Leu858Arg	29
c.2573T > G	p.Leu858Arg	41
ALK_ rearrangments		
	EML4(2)-ALK(20)	
	EML4(13)-ALK(20)	
	EML4(6)-ALK(20)	
	EML4(13)-ALK(20)	
	EML4(13)-ALK(20)	
	EML4(13)-ALK(20)	
	EML4(13)-ALK(20)	
	EML4-ALK	
HERBB2		
c.2593G > A	p.Gly865Arg	3.4
c.2521c > G	p.Leu841Val	15
c.2324_235InsATACG	p.Glu770_Ala771InsAlaTryValMet	13
c.2324_2325InsATAC	p.Glu770_Ala771InsAlaTryValMet	89
c.2263_2264delTTin	p.Leu755Pro	20
BRAF		
c.1801A > G	p.Lys601Glu	15
c.1780G > A	p.Asp594Asn	17
c.1801A > G	p.Lys601Glu	8
c.1742A > T	p.Asn581Ile	12
RET_rearrangments		
	KIF5B(15)-RET(12)	
	KIF5B(24)-RET(11)	
	KIF5B(15)-RET(12)	
	KIF5B(15)-RET(12)	
MET		
Exon 14 skipping		
Exon 14 skipping		
Exon 14 skipping		
ALK		
c.3651G > T	p.Gln1217His	27
PIK3CA		
c.2176G > A	p.Glu726Lys	5
Others		
NTRK1_ rearrangments	TPM3-NTRK	
ROS1_ rearrangments	CD74(6)-ROS1(34)	

Table 4. NGS co-alterations in the clinical implementation cohort.

Case ID	Gene	Mutation Annotation	AF	Clinical Significance
83	KRAS	c.34G > T	66	Pathogenic
	ALK	c.4812C > A		Uncertain significance
89	EGFR	c.2235_2249del	38	Pathogenic
	PIK3CA	c.1633G > A	20	Pathogenic
9	KIF5B(15)-RET(12)			Pathogenic
	HER2	c.2333G > T	5	Unknown
10	KRAS	c.34G > T	18	Pathogenic
	HER2	c.2593G > A	4	Unknown
59	EGFR	c.2303G > T	12	Pathogenic
	KRAS	c.35G > T	22	Pathogenic
112	EML4(13)-ALK(20)			Pathogenic
	MET	c.3335A > T	4	Unknown
	Exon14 skipping			Pathogenic
26	KRAS	c.35G > A	20.3	Pathogenic
	MET	c.3029C > T	44	Conflicting interpretations of pathogenicity
31	KRAS	c.35G > T	158	Pathogenic
	MET	c.504G > T	50	Likely benign
28	EGFR	c.2573T > G	29	Pathogenic
	PI3KCA	c.3140A > G	34	Pathogenic
11	EGFR	c.2573T > G	41	Pathogenic
	PIK3CA	c.1638G > C	19	Pathogenic
109	BRAF	c.1742A > T	12	Pathogenic
	PIK3CA	c.2164G > A	7	Pathogenic

Supplementary Methods

Methods S1. Sanger Sequencing

The PCR amplifications of exons 18, 19, 20 and 21 of EGFR gene were performed. The amplified products were enzymatically purified using FastAP Thermosensitive Alkaline Phosphatase and Exonuclease I (Fermentas, Waltham, MA, USA), sequenced with BigDye Terminator v3.1 Cycle Sequencing Kit (Applied Biosystems, Waltham, MA, USA) and run on the ABI PRISM 3130xl Genetic Analyzer (Applied Biosystems) according to manufacturer's instructions. Variant analysis was carried out with the software Mutation Surveyor 3.24 (Softgenetics LLC, State College, PA, USA).

Methods S2. Fluorescence *in situ* Hybridisation (FISH)

ALK fusions were detected using the Vysis ALK break-apart FISH probe kit following manufacturer instructions (Abbott Molecular, Abbott Park, IL, USA). Only patients that tested negatively for EGFR were tested for ALK fusions.

Abbreviations

AKT1	AKT serine/threonine kinase 1
ALK	ALK receptor tyrosine kinase
BRAF	B-Raf proto-oncogene, serine/threonine kinase
CTNNB1	catenin beta 1
DDR2	discoidin domain receptor tyrosine kinase 2
EGFR	epidermal growth factor receptor
ERBB2	erb-b2 receptor tyrosine kinase 2
ERBB4	erb-b2 receptor tyrosine kinase 4
FBX7	F-box protein 7
FGFR3	fibroblast growth factor receptor 3
FGFR1	fibroblast growth factor receptor 1
FGFR2	fibroblast growth factor receptor 2
KRAS	KRAS proto-oncogene, GTPase
MAP2K1	mitogen-activated protein kinase kinase 1
MET	MET proto-oncogene, receptor tyrosine kinase
NOTCH1	notch receptor 1
NRAS	NRAS proto-oncogene, GTPase
PTEN	phosphatase and tensin homolog
PIK3CA	phosphatidylinositol-4,5-bisphosphate 3-kinase catalytic subunit alpha
STK11	serine/threonine kinase 11
SMAD4	SMAD family member 4
TP53	tumor protein p53
